# CD109 Plays a Role in Osteoclastogenesis

**DOI:** 10.1371/journal.pone.0061213

**Published:** 2013-04-12

**Authors:** Yongqiang Wang, Maayan Inger, Hongwei Jiang, Howard Tenenbaum, Michael Glogauer

**Affiliations:** 1 Matrix Dynamics Group, Faculty of Dentistry, University of Toronto, Toronto, Ontario, Canada; 2 Department of Periodontology, Faculty of Dentistry, University of Toronto, Toronto, Ontario, Canada; University of Medicine and Dentistry of New Jersey, United States of America

## Abstract

Osteoclasts are large multinucleated cells that arise from the fusion of cells from the monocyte/macrophage lineage. Osteoclastogenesis is mediated by macrophage colony-stimulating factor (M-CSF) and receptor activator of nuclear factor-kB ligand (RANKL) and involves a complex multistep process that requires numerous other elements, many of which remain undefined. The primary aim of this project was to identify novel factors which regulate osteoclastogenesis. To carry out this investigation, microarray analysis was performed comparing two pre-osteoclast cell lines generated from RAW264.7 macrophages: one that has the capacity to fuse forming large multinucleated cells and one that does not fuse. It was found that CD109 was up-regulated by>17-fold in the osteoclast forming cell line when compared to the cell line that does not fuse, at day 2 of the differentiation process. Results obtained with microarray were confirmed by RT-qPCR and Western blot analyses in the two cell lines, in the parental RAW264.7 cell line, as well as primary murine monocytes from bone marrow. A significant increase of CD109 mRNA and protein expression during osteoclastogenesis occurred in all tested cell types. In order to characterize the role of CD109 in osteoclastogenesis, CD109 stable knockdown cell lines were established and fusion of osteoclast precursors into osteoclasts was assessed. It was found that CD109 knockdown cell lines were less capable of forming large multinucleated osteoclasts. It has been shown here that CD109 is expressed in monocytes undergoing RANKL-induced osteoclastogenesis. Moreover, when CD109 expression is suppressed *in vitro*, osteoclast formation decreases. This suggests that CD109 might be an important regulator of osteoclastogenesis. Further research is needed in order to characterize the role played by CD109 in regulation of osteoclast differentiation.

## Introduction

Osteoclasts are large multinucleated cells [1], [2], [3] that play an essential role in the extracellular resorption of mineral and organic bone matrix components and regulation of calcium influx in the body [4]. These cells also participate in the pathogenesis of chronic osteolytic diseases such as periodontitis, osteoporosis and rheumatoid arthritis [5], [6]. Osteoclasts arise in the bone marrow from the fusion of hematopoietic cells of a monocyte/macrophage lineage after stimulation by macrophage colony-stimulating factor (M-CSF) and receptor activator of nuclear factor-kB ligand (RANKL) [1], [2], [3], [7]. This process, osteoclastogenesis (OCG), involves the participation and interaction of several biomolecules/cytokines such as IL-1, IL-6, MCP-1, TNF-α, TGF-β, OPG, vitronection, β-integrins, CD44, CD200, DC-STAMP, Sirpα, Tetraspanins, ATP6v0d2, OC-STAMP, TRAF6 and TGF-β [1], [8], [9], [10], [11], [12]. However, there are likely other molecules that play an integral role in OCG that have yet to be identified.

Data from microarray analysis carried out in this laboratory revealed that CD109 is upregulated in the fusing osteoclasts. This novel protein, CD109, is a glycosyl-phosphatidylinositol (GPI)-anchored protein of approximately 170 kDa that belongs to the α2-macroglobulin/C3, C4, C5 family of thioester-containing proteins [13], [14]. It has been found to be expressed in activated platelets and T cells, endothelial cells, leukemic megakaryoblasts, and a subpopulation of CD34 expressing cells [13], [14], [15], [16], [17]. CD109 has been identified as a co-receptor for TGF-β and when it binds to the TGF-β1 subtype, a heteromeric complex is formed with its receptors [18], [19]. The high affinity that CD109 has for the TGF-β receptors, inhibits TGF-β signaling independent of ligand sequestration inducing TGF-β receptor compartmentalization and internalization into caveolae [18], [20]. Several studies have shown that human cells overexpressing CD109 exhibited enhanced proliferation compared with control cells and therefore this molecule is known to be involved in tumourigenesis [21]. In relation to this, it is known that the gene for CD109 is mutated in colorectal cancer [22] and its expression is altered in many other types of malignancies [20], [21], [23], [24], [25], [26], [27], [28] and even in psoriasis [29].

However, to date there are no reports in the literature regarding the expression of CD109 in the monocyte/macrophage lineage or active osteoclasts while its role in osteoclast formation and/or function has not been identified. Therefore, the aim of this study is to describe the role of CD109 in osteoclast formation.

## Materials and Methods

### Establishment of osteoclastogenic cell lines

BglII and EcoRI (underlined) flanked Lifeact-mEGFP was amplified by using a primer pair 5′-GCGCAGATCTATGGGTGTCGCAGATTTGATCAAGAAA-3′and 5′GCGCGAATTCCTATTACTTGTACAGCTCGTCCATGCCGAG-3′. pmEGFP-N1-Lifeact was used as the template. The resulting PCR product was digested with BglII and EcoRI and ligated into the corresponding sites of a retroviral vector pMSCVpuro. The construct and the packaging plasmid pVSV-G were co-transfected into GP-293 cells using FuGENE HD® transfection reagent (Roche Diagnostics GmBH, Mannheim, Germany). The resulting viruses were transformed into RAW264.7 cells (ATCC no: TIB-71, Passage 6) and individual puromycin (final concentration 7 µg/mL) (Sigma-Aldrich, St. Louis, MO, USA) resistant clones were picked up using a standard limiting dilution method. Two different cell lines were established and were named H10 and C8 according to their osteoclastogenic capacity.

### Osteoclast differentiation and TRACP staining

Osteoclastogenesis (OCG) was initiated by plating 5×104 H10 and C8 cells separately in 12-well Falcon® plates, with each well containing a 18CIR Fisherbrand cover glass. The cells were incubated in 2 mL of Dulbecco' Modified Eagle Medium (DMEM, Life Technologies, Grand Island, NY, USA) supplemented with 10% Fetal Bovine Serum (FBS) and antibiotics (164 IU/mL of penicillin G, 50 µg/mL of gentamicin, and 0.25 µg/mL of fungizone) (complete medium). Soluble recombinant RANKL (sRANKL), purified with Glutathione Sepharose 4B resin (GE Healthcare, Piscataway, NJ, USA) from E. Coli BL21(DE3) (Agilent Technologies, La Jolla, CA, USA) transformed with pGEX-4T-1-sRANKL, was added once the cells were placed in the dishes (final concentration 60 ng/mL). Cells were cultured for 4 days, with a change of cell culture medium and sRANKL supplementation on the second day of incubation. At day 4, cells were gently washed twice with pre-warmed PBS (phosphate buffered saline), fixed with 4% paraformaldehyde (PFA) and stained for tartrate-resistant acid phosphatase (TRACP), which is considered a chemical marker of osteoclasts [30], [31]. Briefly, the fixed cells were incubated in a solution of naphthol AS-TR phosphate disodium salt and fast red TR salt (Sigma-Aldrich) in 0.2 M acetate buffer (pH 5.2) containing 100 mM sodium tartrate (Sigma-Aldrich) for 15 min at 37°C. TRACP-stained cells were washed twice with PBS, counterstained with DAPI (diamidino-2-phenylindole, Fluka, Buchs, Switzerland; 0.165 µM in PBS containing 0.1% Triton® X-100) for 10 min and viewed with a Nikon Elipse E1000 microscope.

### Microarray

H10 and C8 cells were plated for two days in 60-mm Falcon®tissue culture dishes at a density of 0.5 × 10^6^ cells/dish in the complete DMEM containing sRANKL (60 ng/mL). Following two days of culture, total RNA was extracted (Qiagen RNeasy Mini kit, Germantown, MD, USA) and the concentration was measured (NanoDrop 1000 Spectrophotometer, Thermo Scientific, Wilmington, DE, USA). Samples were submitted to the Centre for Applied Genomics at Sick Children's Hospital (Toronto, ON, Canada), where RNA quality was examined (Agilent BioAnalyzer) and microarray analysis was performed using the Mouse Gene 1.0 ST array (Affymetrix).

#### Microarray data analysis

Raw data were normalized using a robust multi-array average (RMA) algorithm [32]. In order to identify genes that were expressed differentially, LIMMA (linear models for microarray data) was used [33]. This test is similar to an ANOVA except that the residual standard deviations are moderated across genes to ensure more stable inference for each gene. Genes with false discovery rate (FDR) equal to *p<0.05* and fold change ≥5 were selected as having been up or down-regulated enough to be considered as representing true changes in gene expression. The microarray data has been reported at the Gene Expression Omnibus website and it can be found with the following accession number: GSE43811.

### Validation experiments

#### Isolation of murine bone marrow monocytes and in vitro osteoclastogenesis

Bone marrow-derived osteoclast progenitors or bone marrow monocytes (BMMs) were isolated from 6–12 week old wildtype (WT) mice (SV129/BL6). Mice were sacrificed via carbon dioxide asphyxiation, and the tibia and femora were removed aseptically and dissected free of adherent soft tissue inside a laminar air flow bio-safety cabinet. Bone ends were cut, and the marrow space was flushed out using a sterile 26-gauge needle with Minimum Essential Medium Alpha (α-MEM) (Life Technologies, Grand Island, NY, USA). The flushed marrow was passed through a 20-gauge needle until a homogenous single cell suspension was created. To remove stromal cells and fibroblasts present in the marrow cell suspension, cells were cultured overnight in the complete α-MEM. Non-adherent cells were harvested and cultured for two days in the complete α-MEM supplemented with 20 ng/mL M-CSF (Sigma-Aldrich). Adherent BMMs were harvested with a cell scraper and counted by using a Z1 Coulter Particle Counter (Coulter Electronics, Hialeah, FL, USA). Osteoclastogenesis was induced by seeding the cells (0.5 × 10^6^ cells/dish) in 60-mm Falcon®tissue culture dishes and incubated at 37°C in a humidified atmosphere of 5% CO2 in air.

Bone marrow cells isolation from mice was performed in accordance with the Guide for the Humane Use and Care of Laboratory Animals and the protocol used in this study was approved by the University of Toronto Animal Care Committee.

#### RT-qPCR analysis

At days 0 through 4 or 8 of OCG, total RNA was extracted from RAW264.7 and bone marrow cell cultures (Qiagen RNeasy Mini kit, Germantown, MD, USA). Total RNA (500 ng) was reverse transcribed into cDNA using Superscript II (Invitrogen Life Technologies, Carlsbad, CA, USA) and Oligo-dT18VN primer (ACGT Corp., Toronto, Ontario, Canada). RT-qPCR primer sequences were obtained from the Harvard Medical School Primerbank (Table 1). A 1: 10 cDNA dilution was used for all reactions. Real-time quantitative reverse transcription PCR (RT-qPCR) was performed in 20 µL reactions containing 5 µL of diluted cDNA, 0.4 µL sense and anti-sense primers (stock concentration 5 µM), 10 µL of SsoFast EvaGreen Supermix (Bio-Rad, Hercules, CA, USA) and 4.2 µL DNase/RNase-free H2O using the BioRad CFX96 Real-time System. Each reaction was done in triplicates. PCR conditions were as follows: the enzyme was activated for 30 seconds, initial denaturation was carried out at 95°C for 5 seconds, annealing and extension temperature was set at 60°C for 5 seconds. After 40 cycles, the melting curves of PCR amplicons were obtained with temperature 95°C for 5 seconds, 65°C for 5 seconds, and ranging from 65°C to 95°C with a 0.5°C increase in temperature every 5 seconds. Data were normalized with internal GAPDH and represented as fold-change to day 0, or directly expressed as 2-ΔΔCT [34]. As shown previously, expression of GAPDH did not change under these experimental conditions and could therefore be considered as being usable for normalization of mRNA expression for other genes.

**Table 1:** RT-qPCR Primer sequences.

Gene	Primer sequences		Amplicon size (bp)
GAPDH	Sense (S)	5′-CCTTCCGTGTTCCTACCCC-3′	131
	Anti-sense (AS)	5′-GCCCAAGATGCCCTTCAGT-3′	
CD109	S	5′-TCCCGCTTTCTGGTGACAG-3′	71
	AS	5′-AGGAGATCCACCCCAATAGTC-3′	
Trim30	S	5′-CCACAATTCCAGTATCCTGGTC-3′	83
	AS	5′-GAGTGCCAGCTTTCCGATCC-3′	
Usp18	S	5′-TGCCTCGGAGTGCAGAAGA-3′	206
	AS	5′-CGTGATCTGGTCCTTAGTCAGG-3′	
Lgals9	S	5′-TTACTGGACCAATCCAAGGAGG-3′	105
	AS	5′-AGCTGTTCTGAAAGTTCACCAC-3′	
Irf7	S	5′-TCCAGTTGATCCGCATAAGGT-3′	80
	AS	5′-CTTCCCTATTTTCCGTGGCTG-3′	
Serpinb9	S	5′-GGATGAGTTGCCAGCTAGATTG-3′	161
	AS	5′-TGGACCACATAATGTCTGGTTTG-3′	
Il7r	S	5′-GCGGACGATCACTCCTTCTG-3′	144
	AS	5′-GCATTTCACTCGTAAAAGAGCCC-3′	
Parp14	S	5′-AGCAGTGGATCAGAAAAGACAG-3′	107
	AS	5′- GTCAGCACCATCTCGGATACT-3′	

In order to compare CD109 mRNA expression in different cell lines at the same osteoclast differentiation time-point, H10 and C8 cells (0.25×10^6^ cells/dish) were stimulated with 60 ng/mL sRANKL in the complete DMEM for 4 days in 60-mm Falcon®tissue culture dishes. The medium was changed at day 2 of cell differentiation process. Unstimulated, parallel cultured original RAW264.7 cells were used as the control. Total RNA was extracted every day. Reverse transcription reactions were performed using 1 µg total RNA as the template. RT-qPCR was conducted using methods described above.

#### Western blotting

To evaluate CD109 expression in different stages of osteoclast differentiation at the protein level, freshly isolated BMMs, RAW264.7, H10 and C8 cells were cultured for various days in the complete α-MEM (BMMs) or DMEM (RAW264.7 and its cell lines) containing sRANKL (and M-CSF in the case of BMMs) as described previously. Subsequently, cells were lysed with 200 µL of pre-cooled RIPA buffer (Sigma-Aldrich) supplemented with 1 mM phenylmethylsulfonyl fluoride (PMSF) and 1× protease inhibitor (BD Pharmingen™ Mississauga, ON, Canada) on ice for 5 min and then collected by cell scraping. Cell lysates were centrifuged for 5 min at maximum speed at 4°C to remove cell debris. Protein concentrations were measured using the BCA protein assay kit (Pierce, Rockford, IL, USA). Equal amount of samples were boiled in 1× Laemmli sample buffer for 10 min and reserved for subsequent Western blotting. Samples were subjected to sodium dodecyl sulfate-polyacrylamide gel electrophoresis using a 7.5% polyacrylamide gel, transferred onto nitrocellulose membrane (GE Healthcare) for immunoblotting. The following antibodies were used: goat polyclonal IgG anti-mouse CD109 (S-20, Santa Cruz Biotechnology, Inc., Santa Cruz, CA, USA; 1: 200) followed by horseradish peroxidase (HRP)–conjugated donkey anti-goat IgG (Santa Cruz Biotechnology Inc., 1: 2,000). Both primary and secondary antibodies used for Western blotting were diluted in Tris-buffered saline plus 0.05% v/v Tween-20 (TBS-T) with 5% milk. Immunoreactive protein was detected using chemiluminescence with ECL Plus (GE Healthcare) on exposure to Bioflex MSI film (Clonex, Markham, ON, Canada). Band intensities on scanned films were quantified by densitometry using ImageJ Version 1.41 software and normalized against β-actin levels used as internal loading controls. Primary and secondary antibodies used for the detection of β-actin were monoclonal anti-β-actin (Clone AC-74, Sigma-Aldrich, 1: 8,000) and HRP-linked sheep anti-mouse IgG (Amersham Pharmacia Biotech Ltd., Oakville, ON, Canada; 1: 8,000), respectively.

### Retroviral transduction and characterization of CD109 knockdown macrophage cell lines

A retroviral vector expressing four different small hairpin RNA (shRNA) reagents targeting mouse CD109 driven by the U6 promoter was purchased from OriGene (TG507140, Rockville, MD, USA). The same vector expressing a scrambled sequence (TR30013) was used as the control. The shRNA sequences are shown in Table 2.

**Table 2:** shRNA constructs.

Target	Sequence code	Cell line	Sequence
CD109	GI5044**94**	**94**	5′-TAAGAGATAGCATTGATGAAGTCTACGAT-3′
	GI5044**96**	**96**	5′-TGGTTCCTCCATCAATGGCTTATCTTCAC-3′
	GI5044**97**	**97**	5′-CTATGAACCAAGGAGACAGGCAGTGCGAA-3′
	GI3220**99**	**99**	5′-CCATCTCTCAACTTCACAGCCACTGTGAA-3′
Scrambled shRNA cassette	TR300**13**	**13**	5′-GCACTACCAGAGCTAACTCAGATAGTACT-3′

GP-293 pantrophic packaging cells were plated onto 6-well Falcon®tissue culture plates and cultured in complete DMEM until 40% confluence was reached. The cells were co-transfected with 2 µg shRNA retroviral constructs (CD109 shRNA) as well as 2 µg of the pVSV-G envelope protein-packaging vector using FuGENE HD®transfection reagent. The transfected GP-293 cells were incubated at 37°C for three days, then the virus-containing supernatant was harvested and spun free of cells. The viral supernatant was treated with 65 U/mL of benzonase (Sigma-Aldrich) to degrade residual cellular DNA for 30 min at room temperature. The viral supernatant was passed through a Millex-HA, 0.45 µm syringe driven filter unit, and then used to infect RAW264.7 macrophages. Infected RAW264.7 macrophages were incubated for three days at 37°C. The medium in the dishes was replaced with complete DMEM containing 7 µg/mL of puromycin (Sigma-Aldrich) for cell selection and then cultured for another 2 days. CD109 expression in terms of protein level was examined by Western blot analysis. The two CD109 knockdown cell lines with the lowest CD109 protein level were used for further experiments. Cells expressing non-effective shRNA effects (i.e. no reduction in expression of CD109) and the cells transfected with the empty vector pGFP-V-RS were used for control purposes.

*In vitro* assays of OCG were performed in the knockdown cells as described above. The number of fused nuclei in ten random microscopic fields (20×) was examined manually. A fusion index (FI, #nuclei in the multinucleated osteoclasts/total nuclei per 10 microscopic fields × 100) was calculated for all cell lines. TRACP positive cells with three or more nuclei were judged as multinucleated osteoclasts.

### Statistical analysis

Each experiment was performed in triplicates unless stated otherwise. Statistical analysis was performed using the non-parametric test, the independent-sample Kruskal-Wallis test, due to the small sample size, and a *p*-value of less than 0.05 was considered statistically significant. To compare different cell lines at different time points, a comparison of means was performed and a *p*-value of less than 0.05 was also considered statistically significant. All statistical analyses were performed with the SPSS 12.0 statistics package (SPSS, Chicago, IL, USA).

## Results

### Difference in osteoclast fusion found in established cell lines

**Figure 1:**
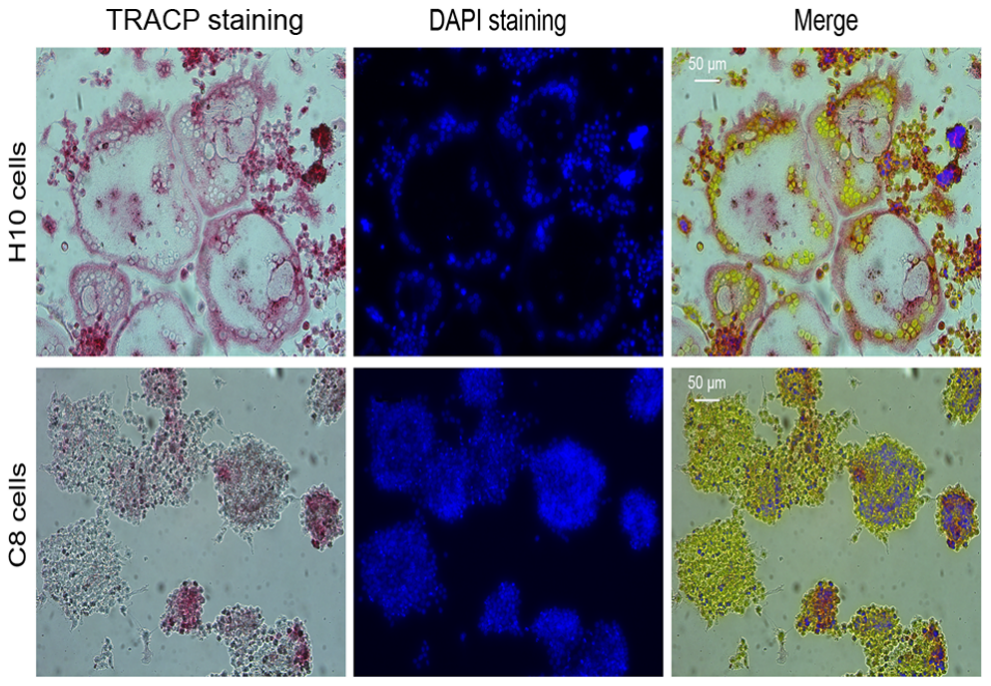
Osteoclast formation potential of H10 cells *vs.* C8 cells. Photomicrograph of TRACP and DAPI stained osteoclasts derived from H10 and C8 cells after 4 days in culture with sRANKL. H10 cells were able to fuse formed large TRACP positive multinucleated cells (FI>60%) while C8 cells did not fuse at all (FI = 0%).

Two different cell lines were derived from the Lifeact-mEGFP stable transfected RAW264.7 cells. The resulting cell lines had different abilities to form osteoclasts in the presence of sRANKL. After culturing these cells for 4 days with sRANKL under the same conditions, TRACP and DAPI staining showed that one of the cell lines, referred to as H10, fused forming large multinucleated osteoclasts while the other cell line, C8, did not fuse, and so no OCG was observed (Fig. 1). Interestingly, despite the fact that both the H10 and C8 cell lines were derived from a highly osteoclastogenic cell line, RAW264.7 cells, the C8 cell line did not retain the ability to form osteoclasts while H10 cells fused forming large multinucleated osteoclasts. This gave the opportunity to determine the basic molecular differences between these two new cell lines that might explain their disparate abilities to undergo OCG.

### Microarray analysis results

Gene expression microarray is an innovative technology that allows the examination and measurement of expression of thousands of genes at a time. [35], [36], [37], [38]. It consists of a number of probes (single stranded cDNA oligonucleotides) containing small amounts of cDNA of a complementary sequence to the mRNA molecule of the gene that it is targeting. The fluorescence level of the dye shows the level at which tagged mRNA molecules hybridize to their complementary probes. When examining this with a scanner, relevant data such as the level of fluorescence of each spot and the level of background noise can be output in a text file and subsequently analyzed [35].

**Figure 2:**
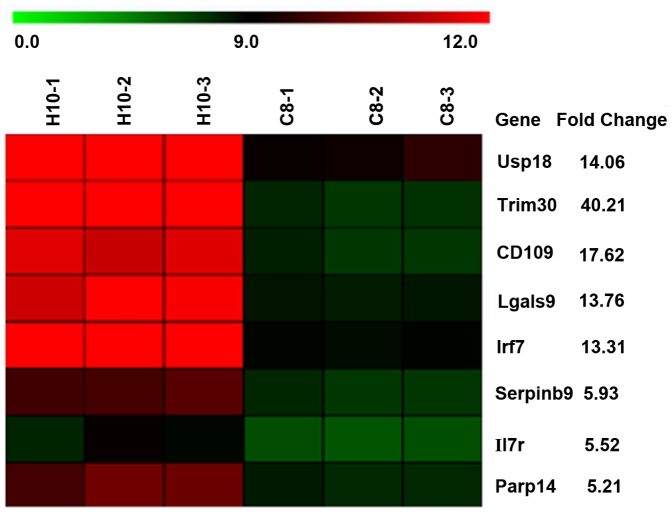
Heatmap of microarray analysis. Total RNA was extracted from H10 and C8 cells undergoing day 2 of RANKL-induced OCG. Microarray analysis was performed with an Affymetrix array and genes with a FDR of 0.01 and fold change over 5 were selected. Heatmap showing 8 randomly selected genes for RT-qPCR confirmation, that have not been reported to be involved in OCG.

In order to evaluate the genetic differences between H10 and C8 cells, both having been derived from RAW264.7 cells, a microarray analysis was performed by extracting total RNA from both derived cell lines. Total RNA was obtained during the early phase of RANKL-induced OCG (day 2 of culture). After analyzing the microarray data under the parameters described above, 42 genes were found to be up-regulated in H10 cells as compared to gene expression demonstrated with C8 cells. Several genes involved in OCG and/or osteoclast function, such as cathepsin K, Prdm1, Tm7sf4 (DC-STAMP), OC-STAMP and Oscar were overexpressed quite robustly at least consistent with 11.87, 6.22, 2.40, 6.02, and 4.48-fold increases respectively. Other genes heretofore not known to play a role in OCG were also found to be up-regulated, such as Usp18, Trim30, Lgals9, Irf7, serpinb9, Il7r, Parp14 and CD109 by 14, 40, 13, 13, 5, 5, 5 and 17-fold, respectively (Fig. 2).

### CD109 expression is up-regulated throughout the days of OCG

**Figure 3:**
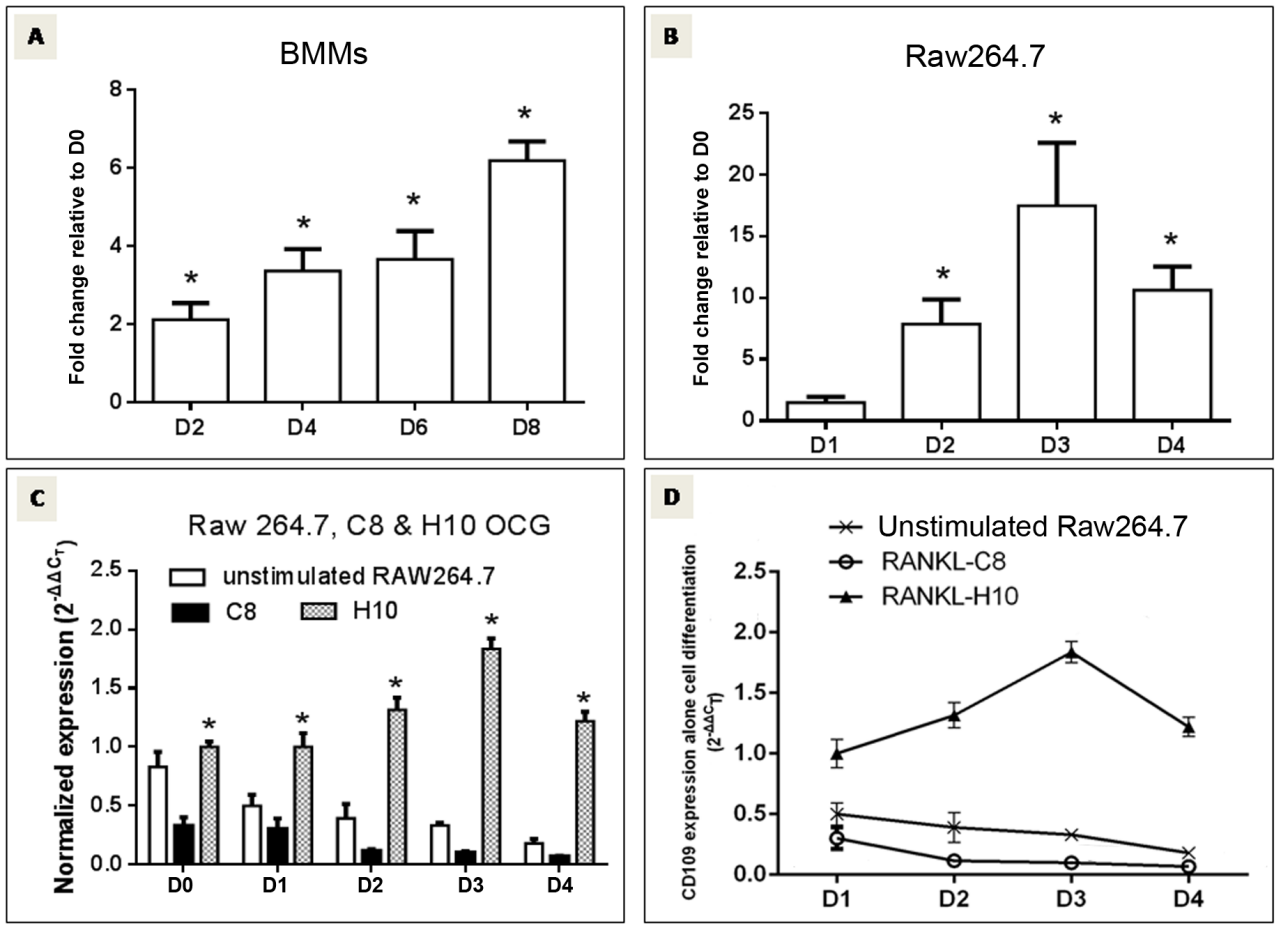
RT-qPCR confirming microarray results. Real-time qPCR analysis was used to quantify gene expression of osteoclast cultures at days 0, 2, 4, 6, 8 for BMMs (A); days 0, 1, 2, 3 and 4 for RAW264.7 cells (B) and day 0, 1, 2, 3 and 4 for H10 and C8 cells (C and D). Day 0 was used as the control in BMMs and RAW264.7 RT-qPCR. CD109 mRNA level in H10 and C8 cells, as well as in unstimulated RAW264.7 cells were expressed as normalized 2^-△△C^_T_. C8 cells were used as the control for H10 cells. There was a statistically significant increase of CD109 mRNA expression over the days of OCG in RAW264.7 and bone marrow monocytes as well as when comparing mRNA expression of CD109 in H10 cells *vs*. both C8 cells and unstimulated RAW264.7 cells at day 1, 2, 3 and 4 of OCG (n = 3, *P<0.05*). Note that CD109 mRNA expression level in C8 cells not only less than that of the H10 cells, but also less than that of the original RAW264.7 cells, regardless of RANKL stimulation (day 1–4 with RANKL, day 0 without RANKL stimulation).

**Figure 4:**
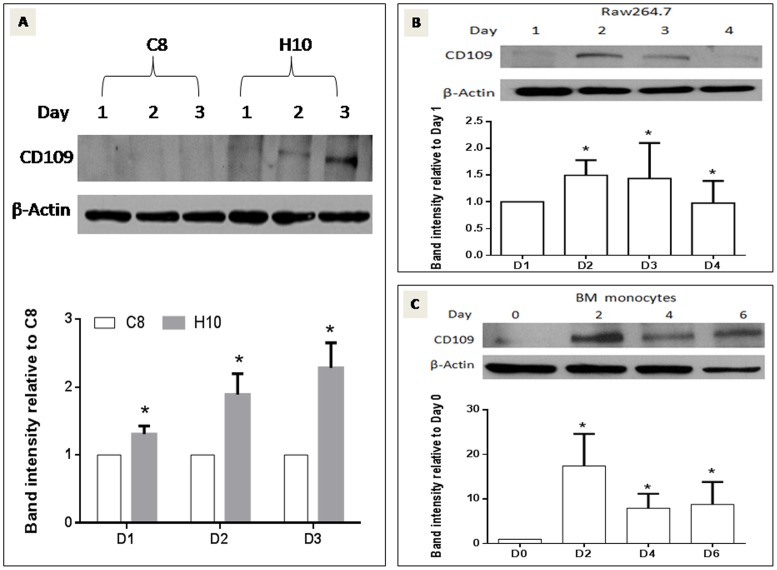
CD109 Protein expression over the days of OCG. Western blot analysis showing that CD109 protein expression is increased in the critical days of osteoclastogenesis in H10 cells, RAW264.7 cells and BMMs (n = 3, *P<0.05*). Band intensity of CD109 expression was normalized with β-actin. Band intensity of C8 cells was used as the baseline for 4A. Day 1 for 4B, and Day 0 for 4C.

After analyzing the microarray data, 8 genes not reported in the literature to be involved in OCG, were selected from the microarray data set and confirmation of mRNA expression was performed by RT-qPCR. Results from the RT-qPCR analysis showed that, of all the tested genes, CD109 was the only one that significantly increased its mRNA expression over the phase of OCG in cells including BMMs and RAW264.7 (n = 3, *P<0.05*) (Fig. 3A and 3B). RT-qPCR analysis of total RNA samples harvested from cells at the same differentiation stages showed that, CD109 mRNA expression level in the sRANKL-stimulated H10 cells at day 1, 2, 3 and 4 was significant higher than that of sRANKL-stimulated C8 and unstimulated original RAW264.7 cells (Fig. 3C). Interestingly, RT-qPCR also showed that, CD109 mRNA expression level in C8 cells not only less than that of the H10 cells, but also less than that of the original RAW264.7 cells, regardless of RANKL stimulation. Increased CD109 mRNA expression was seen only in sRANKL-stimulated H10 cells, but not in sRANKL-stimulated C8 cells and the unstimulated RAW264.7 cells during OCG (Fig. 3D). Confirmation of CD109 protein expression by Western blot analysis was performed in H10 and C8 cells (Fig. 4A) as well as in the original RAW264.7 cells (Fig. 4B) and primary bone marrow monocytes (Fig. 4C). It was found that CD109 protein expression was also increased during OCG in all tested cells (n = 3, *P<0.05*). That CD109 mRNA and protein expression were both demonstrated to be parallel to one another, and further since CD109 was expressed and transcribed in all cell lines as well as in primary murine BMMs, this underscores the rationale for choosing this molecule as the focus of this investigation.

### Fusion capacity of CD109 knockdown cells

**Figure 5:**
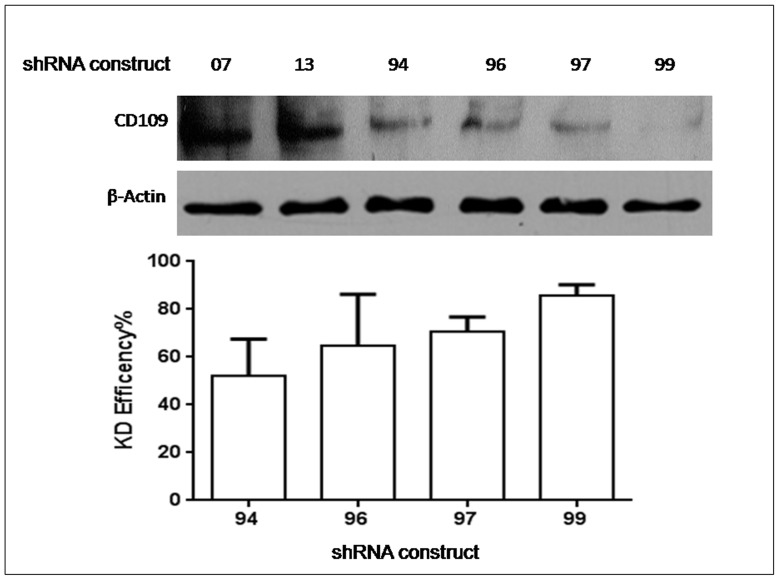
CD109 Knockdown cell lines. Western blot analysis was used to reveal the percentage of protein knockdown per construct used. β-actin was used as the internal loading control. CD109 protein expression in cells expressing a scrambled shRNA (shRNA construct 13) was used as the control. Quantification of the Western blot showed that the higher knockdown efficiency was observed in constructs 97 and 99 when compared to scramble control. Note that 07 is RAW264.7 cells stably transfected with the empty shRNA vector pGFP-V-RS, which was used to harbor gene specific shRNA to knockdown CD109.

Since CD109 was found to be up-regulated during the critical days of OCG, we wanted to characterize the role of this molecule in osteoclastogenesis. In order to do that, CD109 Knockdown cells were originated by using four different shRNA sequences separately and named as 94, 96, 97 and 99 according to the catalog number of each sequence. Once selection of KD cells was done by adding puromycin to the culture, we proceeded to confirm the percentage of CD109 protein knockdown within cells of each shRNA by performing Western blot analysis to assess protein levels of CD109. This revealed 52% of CD109 knockdown in the shRNA 94 cells, 64% of CD109 knockdown in the shRNA 96 cells, 70% of CD109 knockdown in the shRNA 97 cells, and 85% of CD109 knockdown in the shRNA 99 cells (Fig. 5). Because they had the lowest expression of CD109 protein, cells with shRNA 97 and 99 were used for further experiments and cells with a non-effective (scramble) shRNA cassette (shRNA 13) was used as the control.

**Figure 6:**
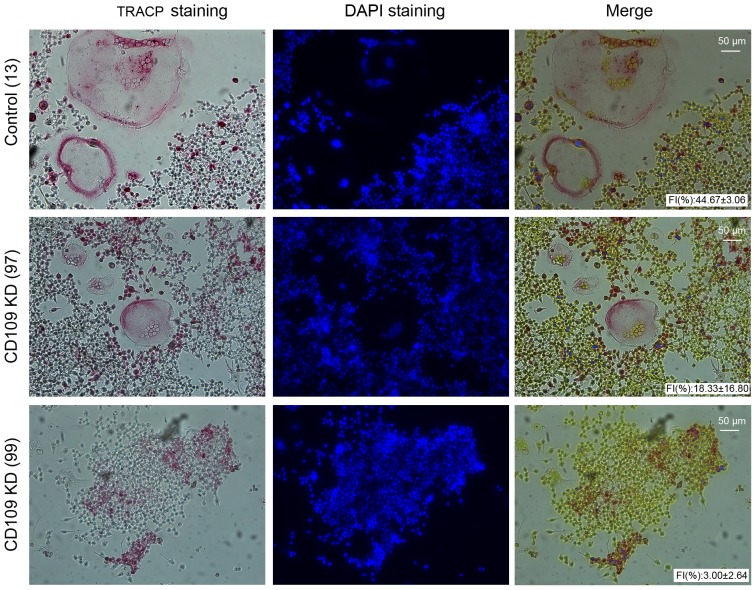
*In vitro* OCG with CD109 knockdown cell lines. RANKL-induced OCG *in vitro* experiments showed decreased fusion efficiency in CD109 KD cells when compared to the negative shRNA control. (n = 3, *P<0.05*).

CD109 KD cell lines were stimulated with sRANKL for 4 days. The last day of culture cells were fixed and TRACP stained. There was a significant decrease noted for fusion efficiency in CD109 KD 97 and 99 cells (18% and 3%) when compared to the control cells containing the scrambled shRNA (44%) (n = 3, *p*<0.05 (Fig. 6). Since cells that underexpressed CD109 were less capable of fusing, it was concluded that CD109 might be an important regulator of OCG.

## Discussion

OCG is a complex process that can be characterized by several different stages of development; stages that rely on the expression of a large number of biomolecules as well as interactions with other cell-types that have not been identified fully [39]. As shown by microarray analysis of pre-osteoclast cell lines during the early phases of OCG, we showed that CD109 was up-regulated in the osteoclastogenic cell line H10. This protein has only been identified recently and has been shown to have characteristics consistent with its being a TGF-β co-receptor. It has been demonstrated that CD109 is expressed and produced in activated platelets and T cells, endothelial cells, leukemic megakaryoblasts, and a subpopulation of CD34 expressing cells [13], [14], [15], [16], [17]. It is considered a marker of tumourigenesis since it has been found that it is overexpressed in several types of malignant cells [28], [40]. However, the expression of CD109 in osteoclasts has not been identified to date and represents a novel finding.

As described in the Introduction, osteoclast differentiation is initiated by simulation of their progenitor hematopoietic cells with M-CSF and RANKL followed by activation of other signaling proteins such as c-Src and Pyk2 [7], [41]. Previous studies have used large scale screening methods such as microarray to identify molecules expressed by RAW264.7 cells and several have focused on proteins participating in RANKL-induced OCG [42]. In order to identify molecules regulated by RANKL, Yang et al. performed microarray analysis in RAW264.7 cells after stimulation with RANKL for up to five days. A number of molecules were identified that appeared to be regulated quite substantially by RANKL induction. The investigators then divided these proteins into 5 different categories depending on their primary molecular activity [43]. The same group of researchers used high-density microarrays to examine gene expression changes in BMMs and RAW264.7 cells after stimulation, at different time-points (2 and 4 days), with sRANKL while using unstimulated cells as controls. After microarray data analysis and confirmation by RT-qPCR, it was found that a specific sequence, named OC-STAMP by the authors, is expressed at a very low level at baseline, while its expression was increased dramatically at both time points during OCG [44]. In the present study, microarray was used in order to determine whether there might be differences in gene expression between two cell types: H10 and C8 cell lines, during early stages of OCG. After two days of treatment with sRANKL (which would ordinarily induce OCG) it was found that the C8 cell line was incapable of fusing. In this manner it was shown that OCG does not occur in the C8 cell line even though it was derived from the osteoclastogenic RAW264.7 cell line and that its absence was associated with an inability of these cells to fuse into new osteoclasts. Alternatively, OCG was induced in the original RAW264.7 (not shown) and H10 cell lines. Subsequently, the C8 cells were used for control purposes (i.e. non-osteoclastogenic) in subsequent experiments that were designed to determine whether there were any unique gene expression patterns that might define cells with the ability to produce osteoclasts. Consistent with other studies, our data showed that genes involved in OCG and/or bone resorption such as cathepsin K, Oscar, GHR, TGF-βr1 and TNF receptor, were up-regulated greatly in the cell line with high fusion efficiency, H10. Additional molecules were up-regulated including Trim30, Usp18 and CD109 (the gene of interest in this particular case), which have, as with CD109, also never been reported to be involved in OCG.

Given that microarray analysis can lead to false positive outcomes, other investigative tools were used to confirm findings demonstrated with this method of assessment. In this regard, measurement of mRNA and protein levels can be performed by RT-qPCR and Western blot, respectively [45]. RT-qPCR is commonly used as the primary validation tool to confirm changes in gene expression demonstrated initially with microarray analysis. Findings obtained by use of RT-qPCR analysis may differ from those produced by microarray analysis due to several factors including biological variability, the quality of RNA harvested from the target tissues or cells, efficiencies of reverse transcriptases and differences in normalization methods for the data produced with various methods of assessment [46], [47]. In the study mentioned above, Yang et al. used RT-qPCR to confirm results obtained with microarray and found that, although OC-STAMP was only up-regulated by 8-fold when measured using microarray the actual amount of up-regulation was in the range of 300-fold when the RNA was analyzed using RT-qPCR. It is also known that measures of gene expression alone do not necessarily mean that there will be concomitant changes in actual synthesis of the protein that is encoded by the mRNA of interest and so protein identification and quantitation is then required; often by use of Western blot analysis [44]. Hence others (Kim et al. 2011) used RT-qPCR and Western blot analysis to evaluate mRNA levels and protein levels of OC-STAMP in RANKL-stimulated BMMs and RAW264.7 cells [8]. In this study, RT-qPCR analysis was carried out for 8 of the genes that were found to be up-regulated in H10 cells, using microarray analysis. Using RT-qPCR analysis it was shown that mRNA for CD109 was expressed in increasingly greater amounts throughout the process of OCG in BMMs, RAW264.7 cells and the H10 cell line. In parallel to the findings demonstrated with mRNA it was also shown, using Western blot analysis, that the relevant proteins, and especially CD109 were produced in increasing amounts over the period of OCG in all of the cell lines that were tested (i.e. RAW264.7, BMMs and H10). Taken together the findings are consistent with the notion that the production of CD109 during OCG requires the presence and activity of RANKL.

In order to determine the role of specific genes that participate in cellular function, a method using shRNA produced inside of the target cells from a DNA construct can be used to interfere (i.e. *silence*) expression of the target mRNA. This is known as “knockdown” [42]. As an example of this, others have demonstrated that when lentiviral constructs expressing a specific shRNA sequence were introduced to BMMs, expression of the protein PARP-1 could be reduced substantially [48]. In light of these previous findings, it was thought that a similar approach could be used to elucidate, at least in part, the biological functions of CD109 in OCG by introducing shRNA sequences for this mRNA in order to develop stable CD109 knockdown cell lines. These cell lines were cultured with sRANKL and it was clear that even after 4 days of culture OCG was reduced/inhibited. These results suggest that CD109 may be an important regulator of osteoclast formation and that monocytes unable to express CD109 are less likely to fuse and form large multinucleated (or functional) osteoclasts.

**Figure 7:**
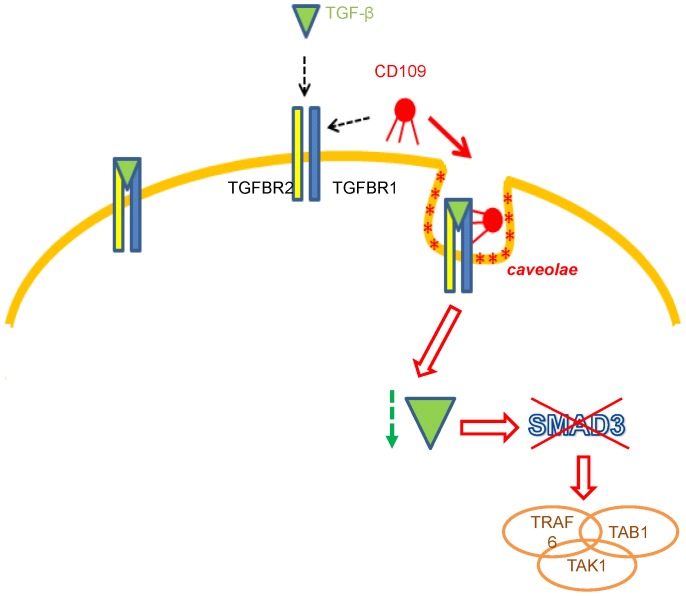
Schematic model of CD109 down-regulation of TGF-β. CD109 down-regulation of TGF-β signaling and inhibition Smad3 signaling, would cause a decrease in the TRAF6-TAB1-TAK1 complex formation resulting in a decrease in osteoclast formation. Up-regulation of CD109 showed an increase in OC formation suggesting that CD109 plays a role in OCG and it might be independent of TGF-β in osteoclasts.

More research needs to be conducted in order to determine the mechanisms by which CD109 affects OCG and whether expression of this molecule is affected by or has effects on expression of TGF-β in osteoclasts given the latter' important role in OCG. Notwithstanding the relative lack of knowledge about this recently discovered protein, it is possible to infer the function of CD109 by using data reported from other published studies. In relation to this, for example, it has been suggested that CD109 might be a co-receptor or decoy receptor for TGF-β that would otherwise bind to cell-associated TGF-β receptors [18], [20], [49]. Studies in human keratinocytes have demonstrated that CD109 promotes TGF-β binding to its receptors located in caveolae with further internalization of this complex, causing down-regulation of TGF-β signaling at the same time [18]. TGF-β plays an essential role in cellular differentiation as well as in OC formation [50], [51], [52], [53]. Its receptors (type I, II and co-receptors) can be internalized in two different ways; clathrin-coated pits and via the caveolae mentioned above. The clathrin-mediated pathway of internalization has been associated with Smad2/3 signaling and receptor recycling [54] while TGF-β receptor localization in caveolae has been associated with down-regulation of Smad2 and Smad3 signaling [18]. Yasui et al. (2011) reported a direct association between Smad2/3 and the TRAF6-TAB1-TAK1 molecular complex which is generated in response to RANKL stimulation and that, when TGF-β signaling was blocked, the TRAF6-TAB1-TAK1 complex cannot form. Moreover, when eliminating Smad3 signaling in osteoclast precursors using gene silencing (i.e. gene knockdown), a significant decrease in RANKL-induced osteoclast differentiation was observed. These authors concluded that the binding of Smad3 to the TRAF6-TAB1-TAK1 complex is crucial for RANKL-induced osteoclastogenic signaling [55]. Bizet et al. (2011) demonstrated that CD109 overexpression inhibits phosphorylation of Smad3 in a caveolin-dependent manner. It was suggested that inhibition of Smad3 by CD109 was due to the putative ability of CD109 to direct the TGF-β ligands to a compartment where their signaling is not effective [18]. When TGF-β signaling is down-regulated by CD109, subsequent inhibition Smad3 signaling occurs which might lead to a decrease in the formation of TRAF6-TAB1-TAK1 complexes. This could then lead to reductions in the formation of active osteoclasts (Fig. 7). According to our results, cells undergoing OCG express more CD109 than those that are inherently unable to form osteoclasts. The same reduction in OCG occurs when the expression of CD109 is reduced using gene silencing. These findings strongly suggest that CD109 plays a critical role in OCG and further that this is not likely related to or reliant upon the TGF-β signaling pathway.

In summary, the current study demonstrated that CD109 is expressed in monocytes undergoing RANKL-induced OCG *in vitro*. Moreover, when CD109 expression is suppressed, osteoclast formation *in vitro* decreases. This suggests that CD109 might be an important regulator of OCG and it appears that it affects this process independent of its proven relationship with TGF-β receptors. Further research is needed in order to characterize CD109 activity in the monocyte/macrophage linage as well as how the expression of this molecule could affect bone metabolism. In developing new treatments for management of diseases characterized by increases in osteoclast formation and/or activity, it is conceivable that, on the basis of the studies reported here, CD109 might represent a novel biotherapeutic target for the creation of new drugs designed to inhibit pathological osteoclast-mediated resorption of bone or other hard tissues. Indeed, regulation of the production of CD109 could be used to reduce OCG and pathological loss of bone, in a more effective and less problematic (i.e. side effects) manner than is available currently.
